# The Effects of Outgroup Threat and Opportunity to Derogate on Salivary Cortisol Levels

**DOI:** 10.3390/ijerph13060616

**Published:** 2016-06-21

**Authors:** Sinthujaa Sampasivam, Katherine Anne Collins, Catherine Bielajew, Richard Clément

**Affiliations:** University of Ottawa, School of Psychology, Vanier Hall, 136 Jean-Jacques Lussier, Ottawa, ON K1N 6N5, Canada; katie.collins@uottawa.ca (K.A.C.); catch@uottawa.ca (C.B.); rclement@uottawa.ca (R.C.)

**Keywords:** outgroup derogation, threat, stress response, intergroup relations, social neuroscience

## Abstract

Perceptions of intergroup threat have been related to both experiences of physiological stress responses and derogation of the outgroup. In this study, a neuroscience perspective was used to investigate the relationship between stress and opportunity to derogate the outgroup, in a threatening intergroup context. Research from a social identity perspective suggests that engaging in outgroup derogation alleviates stress when perceiving an intergroup threat. However, in line with the model of intergroup anxiety, opportunity to derogate could exacerbate the negative connotations of a threatening situation, resulting in more stress. Canadian participants (*N* = 110) were exposed to text describing either discriminatory or favorable comments expressed by Chinese individuals towards Canadians. Half of the participants were given the opportunity to derogate via a bias task. Salivary cortisol was used as a measure of stress and was collected at baseline, post-threat, and post-derogation. As expected, threatening identity led to more stress as evidenced by increased cortisol concentrations. Furthermore, threatened participants who had an opportunity to derogate showed greater cortisol concentrations than those who did not. These results demonstrate a link between stress and the opportunity to derogate, and highlights the value of using biological markers within the intergroup context. Rewrite abstract to remove all the references (they are meaningless because the abstracting services will use the abstract as is but will not provide the references so their presence is useless.

## 1. Introduction

Group membership is used to derive meaning about oneself, and, consequently, motivates identification with positively valued groups and promotes behaviours that advance the group’s interest [1]. Intergroup contact makes salient one’s group membership and highlights the existence of both an ingroup and an outgroup [2,3,4,5,6]. Such contact can, however be considered stressful when it is perceived as threatening or when “one group’s actions, beliefs, or characteristics challenge the goal attainment or well-being of another group” [7] (p. 336). This study explores the physiological stress response via the hormone, cortisol, to experiencing and dealing with an intergroup threat.

How one manages affective experiences may play a role in the relationship between intergroup threats and outgroup attitude [8,9,10]. In particular, displaying negative attitudes about or derogating the outgroup may be a strategy to help individuals cope with any negative emotions and stress that results from the threatening encounters. However, theories in social psychology make different predictions about the functions and consequences of derogation. In this study, we particularly examine research based on theories holding opposing views: (1) Social Identity Theory (SIT: [1]) and (2) The Model of Intergroup Anxiety (MIA: [3]), in view of clarifying the impact of having the mere opportunity to derogate.

## 2. Background

### 2.1. Social Identity Theory Perspective

Social identity, a key component of one’s self-concept, is used to both derive meaning about and to evaluate oneself [1]. Identification with groups that are highly valued in society is said to help group members maintain a positive social identity. Thus, social identity and self-esteem are both influenced by and inextricably tied to the experiences and perceptions of the group to which one belongs [11]. Threats to the ingroup may generate stress and motivate group members to engage in different strategies to alleviate it [12]. Two common intergroup bias strategies that groups use to maintain a positive self-identity are ingroup favoritism and, what is of particular interest here, outgroup derogation [13]. Outgroup derogation refers to discriminating against the outgroup [14]. This may be in the form of treating the outgroup with hostility, making negative evaluations about the outgroup (e.g., [8,15,16,17], and attributing negative traits and responsibility for negative incidents to outgroup members [18].

While outgroup derogation is not as frequent as ingroup favoritism (e.g., [19], it is more prevalent in contexts involving intergroup threat. SIT suggests that outgroup derogation is often used to bolster and protect social identity in the face of a group-based threat. This finding has been observed in a number of intergroup studies. For example, in age-related intergroup contexts, elderly people bolstered their self-esteem by reading negative news articles about young people [20]. Similarly, in gender-related contexts, males who had been exposed to a group threat were more likely to harass females, and subsequently demonstrated increased ingroup identification [21]. Elsewhere, researchers showed participants boxing matches with outcomes that either threatened or supported the value of being American [22]. Participants who identified strongly as Americans were more likely to have reduced collective self-esteem, which subsequently predicted outgroup derogation. Moreover, the amount of derogation displayed was associated with subsequent increases in self-esteem. Similar findings have also been observed within the minimal group paradigm [23,24]. Overall, these studies, in line with SIT, suggest that threatening context elicits engagement in outgroup derogation to bolster self-esteem or identity. Based on this research, we propose that a threatening intergroup experience can evoke feelings of stress, or a sense of having insufficient resources to cope with an event. These feelings can result in a physiological stress response. In order to alleviate these feelings of stress and cope with the situation, threatened people may engage in outgroup derogation. These findings can be further corroborated by looking at studies investigating intergroup relations and physiological stress, where experiencing threats to identity are associated with a physiological stress response [25,26,27,28,29,30,31,32].

Based on SIT, one would expect that experiencing an ingroup threat would lead to physiological arousal and a stress response. Subsequently, an opportunity to engage in outgroup derogation would help reestablish a positive self-identity, thus functioning as a restorative mechanism. This study, rather than studying actual engagement in derogation, the focus was on the impact of having an opportunity to derogate. Previous research has theorized [33] and shown that [34] even an opportunity to derogate or retaliate is considered to be sufficient to deal with perceptions of threat, as it allows participants to deal with a threat if they choose to.

### 2.2. Model of Intergroup Anxiety (MIA)

Theories focusing on the anxiety-inducing component of intergroup contact could provide key insight into the physiological and behavioural consequences of intergroup threats that may differ sharply from models based on SIT. MIA [3] describes the antecedents and consequences of intergroup contact. According to this model, contexts that make the intergroup nature of a contact salient can be anxiety provoking, resulting in various behavioural, cognitive, and emotional consequences. Behavioural consequences include amplifying normative behavioural patterns. That is, when anxiety increases, people are more likely to follow norms more rigidly. Cognitive consequences include information-processing biases. For example, contact and anxiety often increase self-esteem-related concerns, leading to positive ingroup biases when evaluating intergroup differences. In terms of affective consequences, intergroup contexts that elicit anxiety amplify emotional reactions, or more specifically, the arousal resulting from experiencing intergroup anxiety transfers to other elicited emotions. This results in heightened emotional responses during, and evaluative responses following, interactions. For example, based on earlier work [35], the model proposes that interactions resulting in negative emotions or outcomes are likely to show augmented negative evaluations. Thus, it is evident that intergroup contact experiences that result in anxiety can have important consequences and are likely to impact subsequent interactions.

Based on MIA, it can be argued that interactions perceived to be threatening will result in anxiety and thus lead to a stress response. Moreover, given that arousal generated from anxiety amplifies all emotions, an opportunity to derogate the outgroup will make the intergroup context salient again, resulting in more negative emotions and further stress, in contrast with expectations derived from SIT.

### 2.3. Intergroup Threats and Physiological Stress Response

Assessing the divergent predictions of SIT and MIA would be well served by an approach based on the physiological response to derogation. The corresponding framework, social neuroscience, is a growing, interdisciplinary field with an underlying premise that lawful relationships exist between biological systems and social psychological processes [36]. It bridges together biological systems, concepts, and methods with social psychological processes and behaviours (e.g., [37,38,39]) to provide a fuller understanding of their relationship [40]. Employing a neuroscience approach, it is possible to directly measure the variables of interest, without it being subject to controlled processes or self-presentation concerns [36,41,42]. This is of particular importance in this study given that a link between intergroup threats and self-reports of stress have not been consistently found, leading some researchers (e.g., [43] to argue that physiological measures, which circumvent self-presentation concerns, might provide a better evaluation of threat-induced stress.

In studies exploring the physiological underpinnings of intergroup relations, cardiovascular and neuroimaging techniques have most commonly been used (e.g., [41,44,45]). For example, Black adolescents’ vigilance for discrimination was correlated with cardiovascular activity [25], while anticipating interacting with a prejudiced White partner was related to psychological and cardiovascular stress for Latina participants [26]. Black participants who experienced a stereotype threat [27] or described racial stressors [28] showed greater increases in blood pressure compared to that of participants who were in non-threatening situations. Interpersonal interactions with members of devalued or stigmatized group members resulted in cardiovascular responses consistent with threat patterns [27,29]. Finally, a meta-analysis based on 134 samples, in which the studies’ contribution was weighted by sample size, showed that perceived discrimination led to significantly heightened stress responses, generally measured through cardiovascular indicators [30]. Taken together, these studies have shown how various forms of intergroup threat do indeed result in physiological stress responses.

There is a limited number of studies looking at hypothalamic pituitary-adrenal axis (HPA) functioning based on cortisol as a measure of stress. For example, past research has shown how contexts that make identity salient and that are perceived as threatening lead to an increase in cortisol levels [31]. Moreover, using coping strategies that help appraise threatening situations as controllable, along with having a greater sense of optimism, were related to lower cortisol concentrations. Similarly, in a study evaluating threat (sexism) induced stress, women who chronically perceived sexism were more likely to show higher cortisol levels, unless they were placed in a condition in which there was no possibility of sexism [32]. In a study looking at cross-race friendship, participants who found intergroup contact threatening were likely to show increased cortisol reactivity following an interaction with a minority group member; over the course of three cross-race meetings, cortisol reactivity decreased [46]. These studies importantly show that intergroup threats can result in a physiological stress response within an experimental setting and support the use of biological markers in understanding the relationship between threats and outgroup derogation. Even though the evidence bears heavily in favour of the physiological impact of intergroup relations, parallel research on intergroup communication is virtually non-existent (*cf.* [47]).

### 2.4. The Stress Response

Stress can be defined as a sense of having insufficient resources to cope with an event [48]. The extent to which a stressor will generate a physiological response depends on several psychological elements of the context, including whether an event is perceived as unpredictable or uncontrollable or whether there are expectations of negative psychological and/or physiological consequences [49]. According to the allostasis model, a stressor, physiological or psychological in nature, can trigger the secretion of a cascade of stress hormones. This results in a number of physiological changes that help to maintain homeostasis in response to environmental challenges [50,51]. The HPA axis, a major endocrine system, regulates stress responses, principally via the hormone cortisol. Salivary cortisol is typically used to assess HPA functioning and correlates well with serum levels of the hormone [52]. It is a noninvasive method of collecting cortisol that is accomplished readily with minimal interruption and is amenable to serial determinations.

The physiological consequences of chronic stress can result in what has been termed “allostatic load” or “wear and tear” on the body [52]. In a review of the damaging effects of stress, McEwen [53] discussed how allostatic load can increase vulnerability to illness by lowering immune system functioning [54], as well as contributing to the onset and/or progression of diseases such as coronary heart disease [55], hypertension, blood pressure [56], as well as leading to declines in cognitive functioning [57]. Some conditions (Cushing’s syndrome, Alzheimer’s disease, to name a few—see [58]) have been associated with increased HPA activation as measured by cortisol levels while others have been linked to decreased or reduced cortisol secretion—for example, in individuals suffering from post-traumatic stress disorder [59] and in breast cancer survivors [60]. Like other stressors, identity-related stressors have been found to impact health. A number of review articles have found a link between perceived discrimination and negative physical and mental health outcomes (e.g., [31,61,62]). This relationship between discrimination and health may stem from the frequent or chronic activation of the HPA axis.

## 3. The Present Study

The aim of this study was to further understand the function of outgroup derogation by studying its associations with physiological states. Given that previous research has shown how contexts that make identity salient and that are perceived as threatening can result in a physiological stress response [31], studying the physiological impact of dealing with a threatening experience through outgroup derogation would be insightful. Moreover, by using a physiological measure of stress, it may be possible to assess the divergent predictions made by SIT and MIA.

In this study participants read a threatening or non-threatening composition, and subsequently were given an opportunity to derogate or were placed in a control condition. Participants in the control condition engaged in a task that did not require them to think about the threatening composition nor the outgroup. Consequently, it is not possible to know if any change in cortisol concentrations is a reflection of engaging in bias or merely thinking about the outgroup.

### 3.1. Specific Hypotheses

(1) *Threat*. Building on other work (e.g., [31]) and models based on SIT and MIA, we predicted that experiencing a threat to identity would increase salivary cortisol levels while the perception of no threat would not. 

(2) *Opportunity to derogate*. Two different predictions were made about the consequences of having an opportunity to derogate the outgroup. Literature based on SIT and intergroup emotions theory would predict that an opportunity to derogate the outgroup would help participants reestablish a positive identity and deal with the negative emotions, resulting in less stress. On the other hand, MIA theory would predict that the intergroup threat and ensuing anxiety would heighten all emotions. A subsequent opportunity to derogate the outgroup might keep the intergroup nature of the context salient and further augment negative emotions.

### 3.2. Overview

In this study, participants’ cultural identity was the premise for group-based devaluation. An intergroup context was created by describing research on the similarities and differences between Chinese and Canadian cultures. The threatening messages included discriminatory statements that were allegedly made by Chinese individuals against Canadians, and emphasized cultural differences in work habits, family structure, hobbies, and values. Thus, a form of symbolic threat [4,63,64] or threat to group value [65] was created against the Canadian participants. This form of intergroup threat is related to the loss of face, undermining self-esteem or ingroup identity [64] and has been associated with negative outgroup attitudes [7].

## 4. Method

### 4.1. Participants

It was important that participants both clearly belonged to the Canadian group and were aware of the stereotypes associated with being Canadian. Only participants born in Canada, and whose parents were born in Canada, whose mother tongue was English, and whose most frequently spoken language was English were included in the study. In order to ensure that participants did not identify with Chinese groups and consequently discount the threatening composition, participants who identified as Asian Canadian were not recruited. Mother tongue was explained to participants as the first language learned and still most frequently used. A total of 118 undergraduate students, 102 females and 16 males, enrolled in introductory psychology courses, participated in this study. On average, the participants were 19.8 (SD = 4.4) years old. They were recruited through the departmental Integrated System of Participation in Research and received 2% towards their grade for their involvement in this study. The study was advertised to potential participants as looking at the similarities and differences between cognitive and physiological measures. All participants were randomly assigned to the different conditions. The study was approved by the University of Ottawa’s Social Science and Humanities Research Ethics Board (certificate #H05-15-08). In compliance with ethics regulations, participants gave consent prior to the start of the study and were debriefed about the manipulation at the end of the study. 

**Figure 1:**
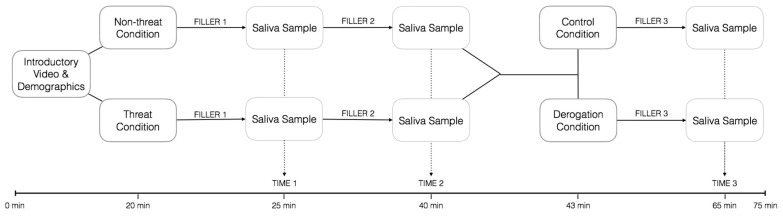
Chart showing flow of participants in the two groups—no threat (**top**) and threat (**bottom**) through each stage of the study.

The design of the experiment as well as the flow of participants through each stage of the experiment appears in Figure 1.

### 4.2. Measures

#### 4.2.1. Salivary Cortisol

Saliva samples were collected three times by asking participants to place an oral swab underneath the tongue for three minutes. An enzyme-linked immunosorbent assay was used to extract cortisol; commercially available kits and the cortisol protocol from Salimetrics Inc. (State College, PA, USA) were used for this purpose. All samples were assayed in duplicate and the mean of the two concentration values employed in the data analyses. Intra-assay variability was 11.40% and inter-assay variability, 17.4%.

#### 4.2.2. Outgroup Derogation

Following the threat manipulation, participants were assigned randomly to one of two conditions. In the control condition, they were asked to write a brief paragraph on time management. In the experimental condition, they were asked for their opinion about the outgroup. This was done so as to minimize social desirability effect by avoiding questions blatantly referring to discrimination against the target group. A series of sixteen vignettes—half depicting socially desirable behaviours (e.g., giving a present), four ingroup members (White) and four in outgroup members (Chinese) and half depicting to the participant socially undesirable behaviours (e.g., fighting), four ingroup members (White) and four outgroup members (Chinese) were presented to the participants. The sex of the actors performing the behaviours varied so that there were an equal number of vignettes containing males and females. Participants were asked to read the vignettes and choose the response that, in their opinion, described the behaviour most accurately. The response alternatives underneath each vignette varied randomly in their level of derogation.

### 4.3. Procedure

Participants meeting the inclusion criteria were tested between 3 PM and 8:30 PM. We selected this time-frame because it corresponds to the time of day when cortisol concentrations are near their lowest value and the diurnal function relating time to cortisol concentration is relatively flat [66]. Participants were asked to attend a lab session during which they were presented with a completely automated online study, with timers programmed to ask participants to provide saliva samples 20 min after they read the threat manipulation and 20 min after having an opportunity to derogate the outgroup.

After providing consent, participants were asked to rinse their mouth with water in order to remove food residue and increase hydration. To ensure that the sample would not be diluted, saliva samples were collected at least 10 min after rinsing. During this 10-min period, participants viewed a video that demonstrated how saliva samples are collected, completed a demographic questionnaire, and reported on a number of variables that could potentially impact the integrity of the cortisol data. Participants responded to a questionnaire inquiring about consumption of any major meal, fluids, or cigarettes one hour before the start of the study, alcohol, and use of medication 12 h prior to the start of the study, any recent major dental work, and whether their gums bled when they brushed their teeth.

Participants were randomly assigned to either the threat or non-threat condition. In both conditions, participants were provided with information from past psychological and demographic studies investigating the similarities and differences between Canadian and Chinese cultures with respect to work habits, family structure, hobbies, and values. These types of threats to group values, belief systems, or worldview, [4,58,59,60] are related to negative outgroup attitudes (e.g., [7]). Thus, to evoke perceptions of threat and negative outgroup reactions in this study, participants in the threat condition were told that the research conducted to date showed that Chinese interviewees had no problem admitting the existence of fundamental differences in values and behaviours, among other things, between Canadians and Chinese. These interviews also revealed a number of discriminatory statements against Canadians. For example, participants read about the interviewees’ perceptions of Canadian culture, “… There is no Canadian culture—that’s why they want to keep the French in—it’s the only thing that separates them from the States and makes them interesting. … Canadian culture is such a joke”. In the non-threat condition, participants were told that Chinese interviewees felt as though the differences between Canadians and Chinese were insubstantial compared to the similarities between the two cultures, and that the interviewees revealed a number of favourable statements about Canadians. For example, “ … Like when there are problems, and two countries, or even two groups, like ethnic, religious, or something like that, are fighting, then Canada tries to bring peace. It makes me proud that they do this”. Consequently, those in the non-threat condition read about social identity enhancement. Participants were then presented with some excerpts from the interview transcripts. All participants were given five minutes to summarize their understanding of the information they had just been presented. The timers were automated so that participants provided a baseline saliva sample (labelled “time 1”) exactly five minutes after finishing reading either version of the composition. Although participants provided the saliva sample after experiencing the threat manipulation, given that peak cortisol concentrations occur roughly 15–20 min following an acute stressor [50], we did not expect there to be an impact of threat on the first sample. We did expect an impact on the second saliva sample (labelled “time 2”), which was collected 20 min following the threat. In between saliva sample collection, participants engaged in a filler task that kept the threat manipulation salient. This task consisted of responding to questions about the composition they read. Participants then either had the opportunity to engage in outgroup derogation or complete the control task. They next engaged in a final filler task which consisted of viewing short videos that kept the intergroup nature of the study salient. They provided a final saliva samples 20 min (labelled “time 3”) after this task. Participants were then debriefed.

## 5. Results

A pilot test was conducted using an independent sample similar to that used in the main study to confirm that the compositions were perceived as expected. Twenty-two participants were assigned to reading either the threatening or the non-threatening composition. They were then asked to rate the composition on 10 items using a 5-point scale labelled at one end as “not at all” (1) and at the other as “completely”. Four items (threatening, offensive, ignorant, disagreeable) constituted the threat scale (α = 0.88) whereas six items (congenial, inoffensive, soothing, pleasant, agreeable, positive) comprised the inoffensive scale (α = 0.96). An independent-samples *t*-test was conducted to compare answers to the threat scale in the threat in threat and non-threat conditions. As expected, those in the threat condition were significantly more likely to perceive the text as threatening (M = 2.98, SD = 1.044) compared to those in the non-threat condition, (M = 1.63, SD = 0.780), t (20) = −3.473, *p* = 0.002. Similarly, an independent-samples *t*-test was conducted to compare perceptions of inoffensiveness in the threat and non-threat conditions. Those in the non-threat condition were significantly more likely to perceive the text as non-threatening (M = 3.68, SD = 0.702) compared to those in the threat condition, (M = 1.67, SD = 1.091), t (20) = 5.236, *p* < 0.001.

We also considered several factors that could influence cortisol secretion such as time since awakening on the saliva collection day, medication use such as birth control, and days since the last menstrual cycle. Across groups, time since awakening averaged from 7.5 to 8.9 h with no group difference (*F* (1,117) = 0.81, *p* = 0.493); of the female participants, only six were using birth control pills, a few in each group, and there was no significant group difference in the number of days since the last menstrual period (*F* (1,101) = 0.32, *p* = 0.81).

Subsequent analyses were conducted using area under the curve (AUC) as the summary measure of change based on trapezoidal rule from ground [67]. After checking for outliers, 9.7% of the data was removed.

**Figure 2:**
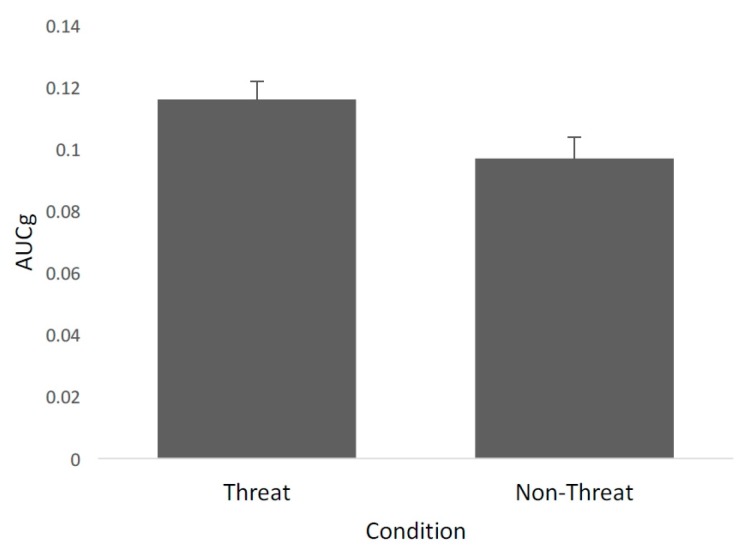
The figure shows the mean AUCg (±SEM) of cortisol concentrations collected at times 1 and 2 as a function of condition—either threat or non-threat.

**Effect of threat on AUCg**. A one-way ANOVA was conducted with threat (present or absent) on the AUC of saliva samples 1 and 2 (see Figure 2). The analysis revealed a significant effect of threat *F* (1,122) = 4.34, *p* = 0.039, *η*^2^ = 0.034). Participants who were threatened (*M* = 0.116, *SE* = 0.006) had a greater AUC compared to those who did not receive a threat (*M* = 0.097, *SE* = 0.007).

**Figure 3:**
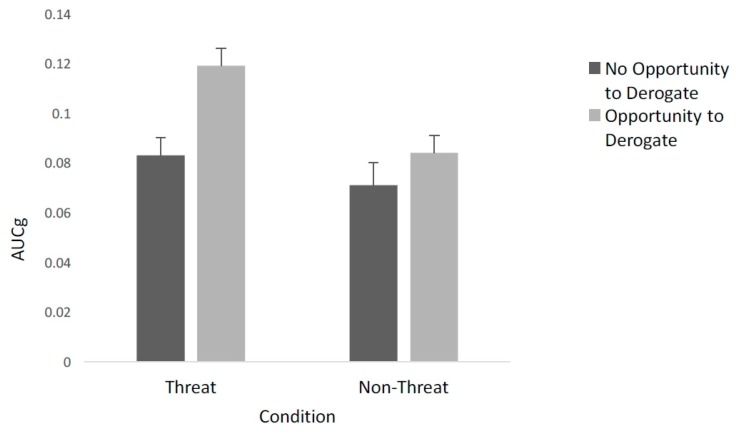
The figure shows the AUC of cortisol samples collected at times 2 and 3 as a function of threat—with the two conditions, threat and non-threat, on the left and right sides respectively as a function of derogation. Dark grey bars represent no opportunity to derogate and light grey bars represent opportunity to derogate.

**Effect of threat and opportunity to derogate on AUC.** A two-way randomized group ANOVA was conducted with both threat (present or absent) and opportunity to derogate (yes or no) on the AUC of saliva samples collected at times 2 and 3 (see Figure 3). The analysis revealed significant main effects of threat (*F* (1,114) = 9.441, *p* = 0.003, *η*^2^ = 0.076.) and opportunity to derogate (*F* (1,114) = 10.53, *p* = 0.002, *η*^2^ = 0.085.) The interaction between threat and opportunity to derogate was not significant, *F* (1,114) = 2.22, *p* = 0.139. However, planned comparisons revealed a significant difference in the AUC of those who had an opportunity to derogate (*M* = 0.119, *SE* = 0.097) and did not have an opportunity to derogate (*M* = 0.083, *SE* = 0.007) in the threat condition, (*F* (1,63) = 10.16, *p* = 0.002, *η*^2^ = 0.139).

## 6. Discussion

The aim of this study was to investigate the relationship between intergroup threats and outgroup derogation in the context of a physiological stress response; the stress hormone cortisol was used as a biomarker to study this relationship. We predicted that experiencing an intergroup threat would result in an increase in cortisol levels. In line with this hypothesis and consistent with other work (e.g., [31,32,46]), participants who were exposed to the threat showed a consistent increase in cortisol levels, indicative of a stress response, compared to those who did not receive a threat. Thus, these findings suggest that group-based threats do indeed incur a stress-related physiological response.

Two different predictions were made about the consequences of having an opportunity to derogate the outgroup. The results indicated that participants who were both threatened and had an opportunity to derogate the outgroup showed an increase in stress levels compared to that of individuals who were threatened but without an option to respond by derogation. In the non-threat condition, there was no difference between participants who had a chance to derogate the outgroup and those who did not. Thus engaging in outgroup derogation increases physiological stress, supporting expectations derived from MIA. This is incongruent with SIT that would posit a decrease in cortisol levels as the opportunity to engage in outgroup derogation would help to reestablish identity and reduce stress.

There are several ways to interpret our findings. First, in line with MIA, it is possible that intergroup contexts elicit emotions like anxiety, resulting in an increase in cortisol levels. An opportunity to derogate the outgroup, which makes the intergroup context even more salient, might amplify other emotions and result in further stress. This would imply that derogation is an inadequate strategy or coping mechanism, given its physiological consequences. Second, an opportunity to derogate the outgroup might have resulted in an increase in cortisol because participants were aware that they were derogating the outgroup. Given that this study is taking place within a Canadian cultural context, which has strong norms about acceptable intergroup behaviour, a chance to derogate the outgroup might increase stress levels. Studies set in cultural contexts with less aversive consequences for outgroup derogation would provide insight on whether an opportunity is sufficient to cause a stress response.

While this study is the first to look at the physiological consequences of outgroup derogation and provides insight into its biological underpinnings, there are some important limitations. Notably, participants did not respond to an actual emotions inventory nor were levels of ingroup identity or outgroup derogation measured. It would be important to document which emotions, including anxiety, are experienced, and how they change with an opportunity to derogate. Given that actual levels of outgroup derogation were not measured, it is unclear whether there were different emotional consequences, and related stress responses, for participants who, when given an opportunity to derogate in response to threat, did and did not engage in derogation. Moreover, the control condition did not require participants to think about the outgroup; it is therefore unclear whether the effects in the outgroup derogation condition resulted because participants were thinking about the outgroup, engaging in outgroup derogation, or engaging in ingroup favouritism. Future research should include a measure of outgroup derogation that makes it possible to delineate these effects.

Despite these limitations, our findings make some key contributions. In addition to replicating the results of previous research showing that a perceived intergroup threat is associated with a stress response as indexed by cortisol concentrations (e.g., [31,32,46]), our data demonstrate the link between outgroup derogation and stress reactivity. While stress responses are often adaptive in the short term, frequent exposure to stressors can result in allostatic load and long-term, negative health outcomes. This can occur through various pathways, for example, when the chronic activation of the HPA axis compromises the feedback mechanism that normally extinguishes the stress response [53].

A growing literature [30,61,62,68,69,70] suggests that perceived discrimination is harmful to both physical and mental health, and contributes to some of the existing health disparities between stigmatized and non-stigmatized groups. Research reviewing the literature on perceived threat and health, has found that perceptions of threat were not only associated with both physiological and psychological stress responses, but also led to more unhealthy (e.g., smoking) and fewer healthy behaviours [30]. It is possible that experiences of discrimination might impact participation in health behaviours which may then contribute to adverse health outcomes. Literature reviewing the effects of perceived racial discrimination on health shows that the negative consequences of perceiving discrimination still exist after socioeconomic status is controlled for [62]. Moreover, the life expectancy disparity between White and Black Americans has only changed from 7 years to 5.1 years between 1960 and 2005 [71], while disparities on other health outcomes (e.g., heart disease) have grown worse. These findings suggest that intergroup experiences such as anticipating and perceiving discrimination contribute to this adverse impact of discrimination on health.

## 7. Conclusions

Our findings provide evidence that outgroup derogation may be a maladaptive response and that frequent exposure to intergroup stressors may have negative and serious consequences. They further pioneer an extension of the field of social neuroscience to intergroup communication [47].
